# Exercise to treat psychopathology and other clinical outcomes in schizophrenia: A systematic review and meta-analysis

**DOI:** 10.1192/j.eurpsy.2023.24

**Published:** 2023-04-25

**Authors:** Daniel Gallardo-Gómez, Michael Noetel, Francisco Álvarez-Barbosa, Rosa María Alfonso-Rosa, Javier Ramos-Munell, Borja del Pozo Cruz, Jesús del Pozo-Cruz

**Affiliations:** 1 Physical Education and Sports Department, Faculty of Education, University of Seville, Seville, Spain; 2 Epidemiology of Physical Activity and Fitness Across the Lifespan (EPAFit) Research Group, Faculty of Education, University of Seville, Sevilla, Spain; 3 Institute for Positive Psychology & Education, Australian Catholic University, Sydney, NSW, Australia; 4 Human Motricity and Sports Performance Department, University of Seville, Epidemiology of Physical Activity and Fitness Across the Lifespan Research Group (EPAFit), Seville, Spain; 5 Biomedical Research and Innovation Institute of Cádiz (INiBICA) Research Unit, Puerta del Mar University Hospital, University of Cádiz, Cádiz, Spain

**Keywords:** Exercise, meta-analysis, psychopathology, schizophrenia

## Abstract

**Background:**

Psychopathology and side effects of antipsychotic drugs contribute to worsening physical health and long-term disability, and increasing the risk of mortality in these patients. The efficacy of exercise on these factors is not fully understood, and this lack of knowledge may hamper the routine application of physical activity as part of the clinical care of schizophrenia.

**Aims:**

To determine the effect of exercise on psychopathology and other clinical markers in patients with schizophrenia. We also looked at several moderators.

**Method:**

MEDLINE, Web of Science, Scopus, CINAHL, SPORTDiscus, PsycINFO, and Cochrane Library databases were systematically searched from inception to October 2022. Randomized controlled trials of exercise interventions in patients 18–65 years old diagnosed with schizophrenia disorder were included. A multilevel random-effects meta-analysis was conducted to pool the data. Heterogeneity at each level of the meta-analysis was estimated via Cochran’s *Q*, *I*^2^, and *R*^2^.

**Results:**

Pooled effect estimates from 28 included studies (1,460 patients) showed that exercise is effective to improve schizophrenia psychopathology (Hedges’ *g* = 0.28, [95% CI 0.14, 0.42]). Exercise presented stronger effects in outpatients than inpatients. We also found exercise is effective to improve muscle strength and self-reported disability.

**Conclusions:**

Our meta-analysis demonstrated that exercise could be an important part in the management and treatment of schizophrenia. Considering the current evidence, aerobic and high-intensity interval training exercises may provide superior benefits over other modalities. However, more studies are warranted to determine the optimal type and dose of exercise to improve clinical outcomes in people with schizophrenia.

## Introduction

Globally, schizophrenia is the third most debilitating mental disorder, behind only depression and anxiety, and this situation is set to worsen with population aging and growth [1]. Psychopathology (i.e., dysfunctional affectivity and positive and negative symptoms) significantly contributes to the poor physical health [2] and long-term disability often observed among patients with schizophrenia [3, 4], thereby increasing the mortality risk in this population group [5]. Current pharmacological approaches are relatively cheap and widely used but offer limited effects on psychopathology [6, 7]. In addition, antipsychotics (i.e., front-line pharmacological treatment for patients with schizophrenia) result in the side effects of weight gain [8] and metabolic syndrome [8, 9]. Thus, non-pharmacological treatments are often used alongside medication in an effort to provide a more comprehensive management of symptoms associated with schizophrenia [10]. Nonetheless, the implementation of non-pharmacological treatments varies across different settings and contexts [11], partially due to a lack of consensus on what is the most effective course of action to manage the symptoms observed in patients with schizophrenia [7, 11].

Exercise may have a wide range of benefits for patients with schizophrenia [12]. For example, by improving cardiorespiratory fitness and metabolic health, exercise may contribute to reducing the physical health problems associated with schizophrenia, such as obesity [13] and diabetes [9], thereby lowering the risk of premature mortality. Other studies have also shown that exercise can positively impact mental health (i.e., depression and anxiety) [14, 15] and cognition [16, 17] in this population group. Thus, exercise is increasingly being recognized as a novel non-pharmacological adjuvant therapy for patients diagnosed with schizophrenia [12].

A number of recent meta-analyses [14–19] have also shown that exercise can significantly improve positive and negative symptoms and affectivity in patients with schizophrenia, yet this evidence should be considered with limitations. First, existing reviews have not considered the clinical setting (i.e., outpatients/inpatients) or the effect of different types of exercises (e.g., aerobic and strength) on many important clinical outcomes, which may limit the understanding of main challenges experienced by people with schizophrenia (e.g., intervention design or poor motivation) in trying to increase this important health behavior [16, 20–22]. Furthermore, the current meta-analytic evidence is also limited by several methodological caveats including failing to account for the existence and length of follow-up of interventions, the different comparator groups reported in the literature, and the use of non-hierarchical analytical techniques when analyzing correlated effect sizes (i.e., different effects sizes sourced from the same study), which may have resulted in biased estimates of the effect of exercise for patients with schizophrenia [23].

Synthesizing the existing evidence using hierarchical meta-analytic techniques while also exploring relevant moderators may help clinicians and decision-makers in considering exercise as part of the treatment array of patients with schizophrenia.

The primary aim of the current study was to review and, through multilevel meta-analytic techniques [24], quantify the current evidence investigating the effects of exercise interventions on psychopathology in patients with schizophrenia. We also investigated if the observed effects were moderated by clinical setting, follow-up, comparison group and type of exercise. A secondary aim of this review was to explore the effect of exercise on a comprehensive array of other clinical outcomes commonly reported in the literature for patients with schizophrenia.

## Methods

### Search strategy

This meta-analysis was pre-registered (PROSPERO 2020 CRD42020180042), and it was conducted according to the Preferred Reporting for Systematic Reviews and Meta-analysis (PRISMA) Statement. Guided by the PICOS framework, we performed a systematic search in the databases MEDLINE, CINAHL, Scopus, WoS, PsycINFO, SportDiscus, and Cochrane Library. The search strategy, dates, and queries are shown in Appendix A of the Supplementary Material. Title/abstract and full-text screening were conducted independently and in duplicate (D.G.-G. and F.A.-B.) with disagreements resolved by discussion or adjudication by a third author (J.P.-C.).

### Inclusion criteria

We searched for and included:

- Participants: Patients 18–65 years old diagnosed with schizophrenia disorder by the Diagnostic and Statistical Manual of Mental Disorders in his fourth or fifth edition (DSM-IV, DSM-V) or by the International Classification of Diseases in his 10th or 11th edition (ICD-10, ICD-11).
- Interventions: Studies that used a specific type of exercise as the main element of the intervention.
- Comparison: Exercise compared with non-exercise treatments or another exercise intervention.
- Outcomes: A range of outcomes such as psychopathology (primary outcome) and quality of life, activities of daily living (ADL) or physical function among others.
- Type of interventions: Randomized controlled trials (RCTs) written in English.

Exclusion criteria were studies reporting mixed interventions with exercise where it was not possible to extract data covering a pure exercise intervention group (e.g., cognitive therapy with exercise) and patients with another additional serious illness (e.g., bipolar disorder and type II diabetes).

### Data extraction

We developed a data extraction spreadsheet. Two authors extracted information independently and in duplicate for each included article. The rest of the authors checked the extracted data corresponding to trial participant’s features (sample size, age, sex, clinical setting, medication type, and doses), descriptive statistics (pre- and post-sample size, means, standard deviations, and standard errors), outcome description (and timepoint), and if applicable, follow-up mean, and standard deviation. Disagreements were resolved by consensus between all authors; and if no agreement could be reached, a third research-independent reviewer was asked. If information was missing, the corresponding author was requested to supply the information or data for inclusion in the analyses.

All outcome data assessed, and their evaluation tools are listed in Appendix B of the Supplementary Material.

### Data synthesis

We conducted a three-level meta-analysis that allowed us to nest effect sizes within studies [23]. Compared with more traditional meta-analytic approaches and based on previous methodological work, this model produces powerful, unbiased, and precise effect size and between-study heterogeneity estimates than simply selecting or averaging effect sizes within studies [23]. However, if a study had multiple intervention conditions, we extracted all exercise-based intervention, but not the other active treatments (e.g., cognitive therapy). Similarly, we extracted all comparison conditions that were not active interventions.

We used the ‘*metafor*’ and ‘*compute.es*’ packages in R [25] to calculate standardized mean differences (Hedges’ *g*) using all available data. Omitting trials with some missing data leads to biased effect size estimates, so when mean and SD were not available, we used other statistics (e.g., confidence intervals and *p*-values) or imputation as described in the guidelines from the Cochrane Handbook [26].

We conducted meta-analyses using the ‘*metaSEM*’ and ‘*msemtools*’ packages in R [25]. We conducted a series of random-effects, multilevel meta-analyses (one for each outcome), where effect sizes were nested within studies. Heterogeneity at each level of the meta-analysis (within and between studies) was estimated via Cochran’s 𝘘, *I*^2^, and *R*^2^ [27, 28]. This meta-analysis model was applied because it was assumed that the observed estimates of treatment/intervention effects vary within/between studies because of real differences in the intervention effects as well as sampling variability [29]. Based on recent evidence [4], we conducted an additional random-effects subgroup meta-analysis model to explore the differential effects of exercise on positive and negative psychopathological symptoms. We then conducted moderation analyses for the following variables: clinical setting (inpatient vs. outpatient), intervention group (type of exercise), control group (type of comparison group), and follow-up (post-test outcomes vs. follow-up). Statistical significance was accepted at a level of *P* < 0.05.

### Risk of bias for individual studies

To assess the risk of bias within studies, we selected the Physiotherapy Evidence Database (PEDro) scale score. The PEDro score is a scale developed to be used in evaluating interval validity and presenting statistical analysis to support clinical evidence-based practice, which allowed us to determine methodological quality and assess risk of bias [29–31]. It presents 10 items. If the study presents the evidence quality indicator, it was assigned 1, and if it did not present it, it was assigned 0. When the score of an article was not shown in the PEDro website, three reviewers (D.G.-G., F.A.-B., and R.M.A.-R.) agreed on a rating of this study following the criteria stipulated by the PEDro scale.

We conducted a sensitivity analysis to assess whether biases identified by PEDro account for variance in the overall effect estimates. To this end, the methodological quality domains included in the PEDro scale (allocation concealment, blinded participants, blinded personnel, blinded assessors, incomplete outcome data, selective outcome reporting, other bias, and overall risk of bias) were entered as moderators in the model conducted for each outcome.

### Risk of bias across studies

We assessed publication bias separately for each outcome. To do this, we created three-parameter selection models (3PSMs) [32], which are more sensitive and specific to the assessment of publication bias than others [33]. They identify whether studies are more likely to be published when significant, and a significant 3PSM likelihood test indicates the presence of publication bias. To quantify the strength of the publication bias, we conducted sensitivity analyses described by Mathur and VanderWeele [34], when results for that outcome were significant. These analyses identify the degree of selection pressure – i.e., the increased likelihood of publication for significant versus nonsignificant studies – needed to explain pooled effects (*s*-value).

### Certainty assessment

The Grading of Recommendations, Assessment, Development and Evaluations (GRADE) system was used to rate the quality of evidence presented in the meta-analyses, and was applied to each outcome because the quality of evidence often varies between outcomes. Because the included studies are randomized controlled trials, it starts with the maximum score (“high quality of evidence”) and as the quality of evidence criteria were applied (risk of bias, imprecision, inconsistency, and publication bias), the score dropped if any outcomes failed to meet criteria for certainty. Three authors graded the evidence from “high” to “very low.” We used the PRISMA statement checklist to provide transparent, complete, and accurate reporting of this systematic review [35].

## Results

### Included studies

**Figure 1.**
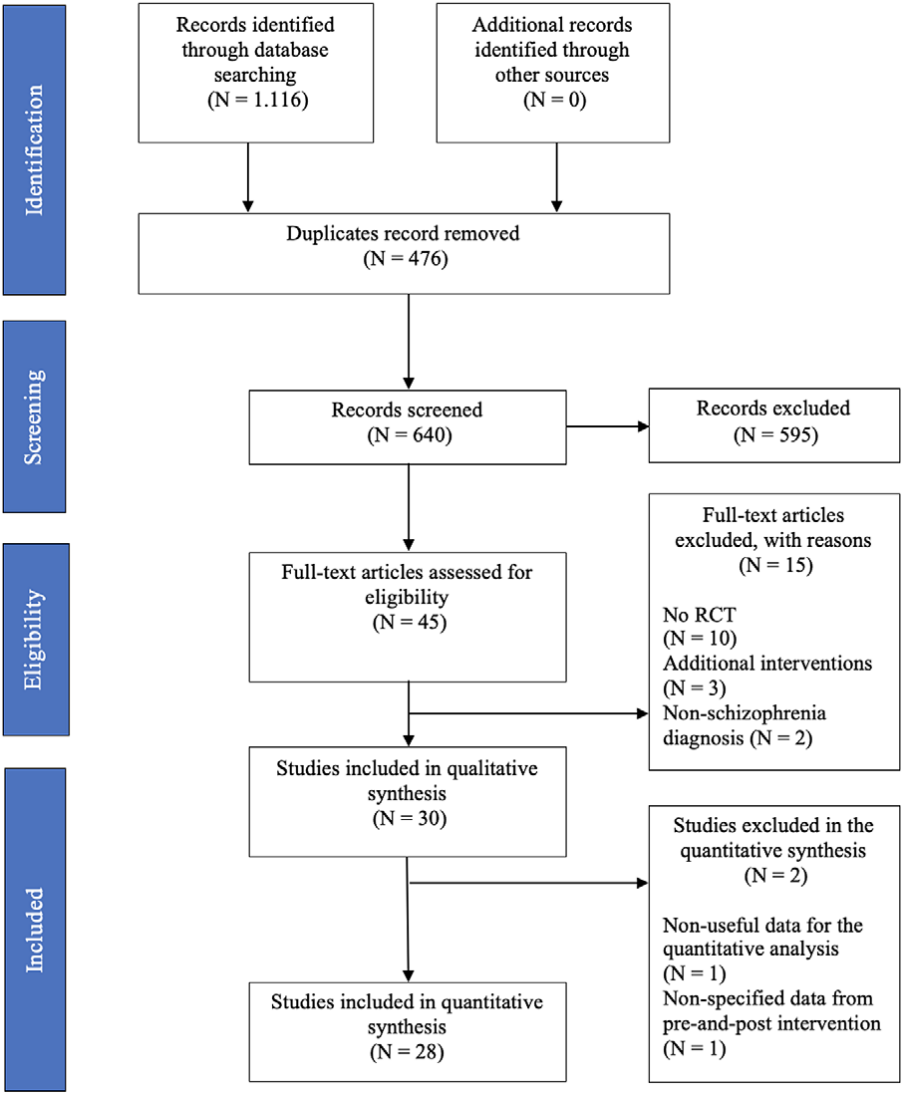
PRISMA flowchart of studies selection applying eligibility criteria.

The systematic search returned 1,116 scientific studies. After removing duplicates and applying the inclusion criteria, 28 studies were selected for inclusion in the meta-analysis. The citations for these articles are included in Appendix B of the Supplementary Material. The complete selection process can be seen in the PRISMA flow diagram (Figure 1). From the final included studies, a total of 332 effect sizes were extracted for 11 outcomes including schizophrenia psychopathology (*N* = 68), quality of life (*N* = 34), anthropometric and body composition (*N* = 34), cardiorespiratory fitness (*N* = 29), ADL (*N* = 16), cognitive function (*N* = 65), muscular strength (*N* = 10), physical function (*N* = 22), physical health biomarkers (*N* = 41), psychological biomarkers (*N* = 9), and stress and anxiety (*N* = 4). Interventions that were directly compared were aerobic exercise versus treatment as usual (*k* [i.e., number of studies] = 10), mind–body interventions (e.g., yoga or tai-chi) versus treatment as usual (*k* = 5), concurrent training (i.e., combination of resistance and aerobic exercises) versus treatment as usual (*k* = 2), high-intensity interval training (HIIT) versus video games (*k* = 2), resistance exercise versus video games (*k* = 1), sports’ games versus treatment as usual (*k* = 1), aerobic exercise versus resistance exercise (*k* = 1), aerobic exercise versus occupational therapy (*k* = 1), resistance exercise versus occupational therapy (*k* = 1), video games versus occupational therapy (*k* = 1), aerobic exercise versus sports’ games (*k* = 1), body-oriented psychological therapy versus treatment as usual (*k* = 1), and resistance training versus concurrent training (*k* = 1). Only two studies reported follow-up data at 3 and 6 months after the intervention.

### Participants’ characteristics and study design parameters

Studies characteristics are shown in Appendix C of the Supplementary Material The year of publication of the included trials vary between 2005 and 2020. In those studies, 1,877 patients participated, and 1,460 were analyzed (34.32% females). Twenty-two studies reported the age of the patients (mean = 39.06, SD = 9.32). Of those patients who were analyzed, 1,124 were outpatients (i.e., non-institutionalized/community-dwelling; 76.79%; *k* = 21), and 336 were inpatients (i.e., institutionalized; *k* = 8). All patients were medicated with antipsychotic treatment based on a variety of drugs (e.g., olanzapine, risperidone, or haloperidol) for at least 6 months. Twenty-five trials reported information about the supervision of their interventions, and the qualification of the trial personnel, which ranged from certified physical trainers to physiologists, physiotherapists, nurses, and researchers. Interventions presented a median dropout rate of 21% (range = no dropouts to 42%), which was highly sample-dependent.

### Psychopathology

**Figure 2.**
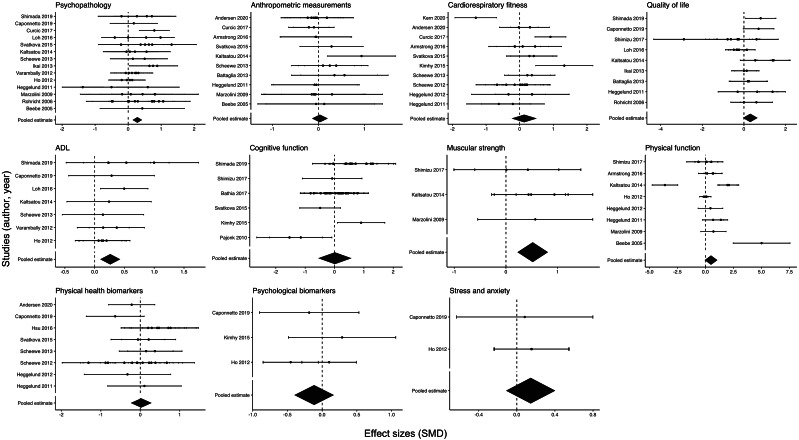
Forest plot showing all study-specific effect sizes and pooled estimates for each analysed outcome. *Note.* Studies are ordered according to their publication year.

Exercise significantly improved the psychopathology of people with schizophrenia (*k* = 14; *N* = 56; *g* = 0.28, [95% CI 0.14, 0.42]). These effect sizes and those corresponding to the rest of the selected outcomes are illustrated in the plot represented in Figure 2. Our subgroup analysis showed that exercise had significant effects on negative symptoms (*N* = 21; *g* = 0.65; [95% CI 0.53, 0.78]) but not on positive symptoms of psychopathology (*N* = 35; *g* = −0.05; [95% CI –0.15, 0.06]) (Table 1).

**Table 1.** Random-effects subgroup meta-analysis results (*N* = 56).

Symptoms	*N*	Hedges’ *g*	SE	*z-*Value	*P*	95% CI lower bound	95% CI upper bound
Positive	35	–0.05	0.05	–0.83	0.405	–0.15	0.06
Negative	21	**0.65** *	0.06	10.20	<0.001	0.53	0.78

* Statistically significant effect size (i.e., *P-value* is below 0.05 and 95% CI does not included the zero)

Initially, it was *Q* **(55)** because of the degrees of freedom of this statistic (number of studies included in this outcome (56) – 1). However, we consider substitute that just using *Q* for a better understanding of the reader and simplicity. So, use (*Q*= 130.05, *P*< 0.001), with heterogeneity (via *I*^2^) at level 2 (between studies) of 52.26% and at level 3 (within studies) of 5.92%. Whether or not patients were inpatients significantly moderated the observed effects ( 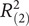 = 25.99%, 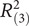 = 100.00%, *P* < 0.001) with stronger results in outpatients (*g* = 0.43, [95% CI 0.29, 0.57], *k* = 9, *N* = 38) than in inpatients (*g* = −0.05, [95% CI –0.29, 0.19], *k* = 4, *N* = 12). Comparison group also significantly moderated effects ( 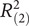 = 21.67%, 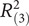 = 100.00%, *P* = 0.03) with exercise leading to greater symptom reduction versus treatment as usual (*g* = 0.29, [95% CI 0.16, 0.42], *k* = 11, *N* = 45) and occupational therapy (*g* = 0.49, [95% CI 0.16, 0.81], *k* = 2, *N* = 8), but not in the one study that used video games as a comparison (*g* = −0.61, [95% CI –1.19, −0.03], *k* = 1, *N* = 3) (Table 2).

**Table 2.** Moderation effects on study outcomes.

Outcome	Moderator	*k*	*N*	Estimate (95% CI)	SE	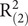	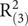	*P*
Psychopathology	Baseline ( 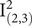 : 0.52, 0.06)	14	56	0.28 (0.14, 0.42)	0.07			
	Clinical setting	14	56			0.26	1.00	0.004*
	Inpatients	4	15	–0.05 (−0.28, 0.19)	0.12			
	Outpatients	10	41	0.43 (0.29, 0.57)	0.07			
	Comparison group	14	56			0.22	1.00	0.008*
	Occupational therapy	2	8	0.49 (0.16, 0.81)	0.17			
	Treatment as usual	11	45	0.29 (0.16, 0.42)	0.07			
	Video games	1	3	−0.61 (−1.19, −0.03)	0.30			
Quality of life	Baseline ( 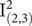 : 0.11, 0.59)	9	28	0.30 (−0.05, 0.65)	0.18			
	Clinical setting	9	28			0.17	0.88	0.004*
	Inpatients	3	7	0.89 (0.52, 1.26)	0.19			
	Outpatients	6	21	−0.07 (−0.36, 0.23)	0.15			
	Intervention group	9	28			0.31	1.00	0.012*
	Aerobic exercise	3	6	0.80 (0.45, 1.14)	0.18			
	High-intensity interval training	1	3	0.77 (0.27, 1.28)	0.26			
	Light–moderate aerobic exercise	3	16	−0.24 (−0.41, −0.07)	0.09			
	Yoga	2	3	0.23 (−0.18, 0.64)	0.21			
	Comparison group	9	28			0.11	0.44	0.14
	Follow-up	9	28			0.90	0.08	0.008*
	No	7	26	0.22 (−0.12, 0.55)	0.17			
	Yes	2	2	1.11 (0.46, 1.77)	0.33			
Anthropometric measurements and composition	Baseline ( 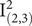 : 0.00, 0.06)	9	20	0.10 (−0.10, 0.30)	0.10			
	Clinical setting	9	20			0.00	0.84	0.24
	Intervention group	9	20			0.00	1.00	0.32
	Comparison group	9	20			0.00	1.00	0.55
Cardiorespiratory fitness	Baseline ( 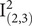 : 0.00, 0.47)	10	28	0.13 (−0.24, 0.51)	0.16			
	Clinical setting	10	28			0.00	0.80	0.01*
	Inpatients	3	6	0.21 (−0.16, 0.50)	0.14			
	Outpatients	7	22	0.31 (0.02, 0.61)	0.15			
	Intervention group	10	28			0.00	1.00	0.051
	Comparison group	10	28			0.00	0.00	0.19
ADL	Baseline ( 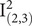 : 0.00, 0.04)	7	13	0.26 (0.09, 0.43)	0.09			
	Clinical setting	7	13			0.00	1.00	0.14
	Intervention group	7	13			0.00	1.00	0.42
	Comparison group	7	13			0.00	0.00	0.72
Cognitive function	Baseline ( 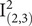 : 0.21, 0.71)	6	47	0.01 (−0.55, 0.58)	0.29			
	Intervention group	6	47			0.00	0.18	0.88
	Comparison group	6	47			0.00	0.15	0.49
	Follow-up	6	47			0.10	0.00	0.11
Muscular strength	Baseline ( 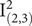 : 0.00, 0.00)	3	6	0.52 (0.22, 0.81)	0.15			
	Clinical setting	3	6			0.00	0.00	0.43
	Intervention group	3	6			0.00	0.00	0.64
	Comparison group	3	6			0.00	0.00	0.35
Physical function	Baseline ( 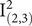 : 0.91, 0.00)	8	17	0.50 (−0.07, 1.06)	0.29			
	Clinical setting	8	17			0.00	0.00	0.96
	Intervention group	8	17			0.06	0.00	0.88
	Comparison group	8	17			0.06	0.00	0.49
	Follow-up	8	17			0.06	0.00	0.11
Physical health biomarkers	Baseline ( 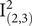 : 0.37, 0.17)	7	25	0.05 (−0.24, 0.33)	0.14			
	Clinical setting	7	25			0.10	0.07	0.51
	Intervention group	7	25			0.29	1.00	0.13
	Comparison group	7	25			0.75	0.00	0.036*
	Occupational therapy	3	10	0.34 (−0.07, 0.75)	0.21			
	Treatment as usual	3	13	−0.24 (−0.66, 0.18)	0.22			
	Video games	2	2	−0.09 (−0.97, 0.79)	0.45			
	Follow-up	7	25			0.00	0.54	0.52
Psychological biomarkers	Baseline ( 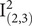 : 0.26, 0.00)	3	4	−0.12 (−0.40, 0.17)	0.15			
	Clinical setting	3	4			0.36	0.00	0.30
	Intervention group	3	4			1.00	0.00	0.20
Stress and anxiety	Baseline ( 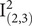 : 0.00, 0.00)	2	3	0.14 (−0.11, 0.40)	0.13			
	Intervention group	2	3			0.00	0.00	0.98

*Note:* Clinical setting moderated whether patients were inpatients or not. Intervention group moderated for the type of exercise prescribed. Comparison group moderated for what was giving to the control condition. Follow-up moderated for whether a post-intervention evaluation was carried out or not. If not all the moderators appeared in a variable, it could mean that (a) the studies that provided the data for them did not have that moderator in common; (b) all the included studies of the same outcome included the same class of a specific moderator; or (c) were removed from the meta-analysis model because of the great amount of uncertainty that introduced.

*k*, number of studies; *N*, number of effects from those studies; SE, standard error.

* *P*-value < 0.05.

In sensitivity analyses, only allocation concealment significantly moderated the results ( 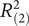 = 5.20%, 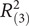 = 100.00%, *P* = 0.03). Higher methodological rigor tended toward stronger effects: studies with adequately concealed allocations demonstrated bigger effect sizes (*g* = 0.35, 95% CI [0.21, 0.49], *k* = 9, *N* = 46) than those for which allocation could have been overlooked (*g* = −0.01, [95% CI –0.29, 0.28], *k* = 5, *N* = 10). The 3PSM likelihood ratio test suggested that these results are unlikely to be influenced by publication bias ( 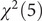 = 6.31, *P* = 0.27). The estimate of the *s*-value indicated that no amount of publication bias could eliminate this effect (lower bound of *s*-value CI: 4.47 times as likely).

### Secondary outcomes

Moderation analyses pertaining to the main and secondary outcomes of this meta-analysis are presented in Table 2.

#### Quality of life

There was no significant overall effect of exercise on quality of life (*k* = 9; *N* = 28; *g* = 0.30, [95% CI −0.05, 0.65]). Clinical setting of interventions significantly moderated effects ( 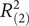 = 17.28%; 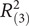 = 87.77%; *P* = 0.004) with larger effects in inpatients (*g* = 0.89, [95% CI 0.52, 1.26], *k* = 3, *N* = 7). The type of exercise also significantly moderated effects ( 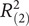 = 31.37%; 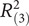 = 100.00%; *P* = 0.012) with significant results for HIIT (*g* = 0.77, [95% CI 0.27, 1.28], *k* = 1, *N* = 3) and aerobic exercise (*g* = 0.80, [95% CI 0.45, 1.14], *k* = 3, *N* = 6) showing large effect sizes. Conversely, light–moderate aerobic exercise had a negative association with quality of life (*g* = −0.24, [95% CI –0.41, −0.07], *k* = 3, *N* = 16). Yoga had a nonsignificant impact (*g* = 0.23, [95% CI –0.18, 0.64], *k* = 2, *N* = 3). Finally, if patients were evaluated at follow-up or not also significantly moderated the observed effects ( 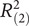 = 89.67%; 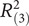 = 7.80%, *P* = 0.008) with significant results for the studies in which patients were analyzed at follow-up (*g* = 1.11, [95% CI 0.46, 1.77], *k* = 2, *N* = 2) but not significant otherwise (*g* = 0.22, [95% CI –0.12, 0.55], *k* = 7, *N* = 26) (Table 2).

In sensitivity analyses, results showed none of the risk of bias domains moderated effects. The 3PSM likelihood ratio test suggested that these results are unlikely to be influenced by publication bias ( 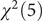 = 2.42, *P* = 0.79).

#### Anthropometric measurements and body composition

Our meta-analysis revealed no effect of exercise on anthropometric measurements and body composition of patients with schizophrenia (*k* = 10; *N* = 34; *g* = 0.04, [95% CI –0.14, 0.22]). None of the moderators assessed were significant (Table 2).

In sensitivity analyses, results showed none of the risk of bias domains moderated effects. The 3PSM likelihood ratio test suggested that these results are likely to be influenced by publication bias ( 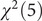 = 14.43, *P* = 0.01).

#### Cardiorespiratory fitness

The pooled effect of exercise on cardiorespiratory fitness was not significant (*k* = 10; *N* = 28; *g* = 0.13, [95% CI –0.24, 0.51]). Clinical setting of interventions significantly moderated the observed effects ( 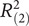 = 0.00%; 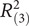 = 79.90%; *P* = 0.01), with results being only significant for outpatients (*g* = 0.31, [95% CI 0.02, 0.61], *k* = 7, *N* = 22) (Table 2).

In sensitivity analyses, only overall risk of bias score significantly moderated results ( 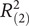 = 0.00, 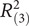 = 0.92, *P* = 0.03) with higher rigor tended toward stronger effects: studies with low overall score of risk of bias demonstrated bigger effect sizes (*g* = 0.91, 95% CI [0.42, 1.41], *k* = 1, *N* = 1) than those where a high risk of bias score were obtained (*g* = 0.10, 95% CI [−0.18, 0.38], *k* = 7, *N* = 24). The 3PSM likelihood ratio test suggested that these results are unlikely to be influenced by publication bias ( 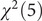 = 9.51, *P* = 0.09).

#### ADL

Exercise significantly improved the ADL of patients with schizophrenia (*k* = 10, *N* = 16; *g* = 0.26, [95% CI 0.09, 0.43]). None of the moderators assessed were significant (Table 2).

In sensitivity analyses, incomplete outcomes significantly moderated results ( 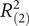 = 0.00, 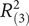 = 1.00, *P* = 0.047) which demonstrated that in the studies which there were more data outcomes, the effect sizes were bigger (*g* = 0.47, 95% CI [0.22, 0.72], *k* = 4, *N* = 6) where the incomplete data outcomes was notorious (*g* = 0.16, 95% CI [0.01, 0.32], *k* = 3, *N* = 7). The 3PSM likelihood ratio test suggested that these results are likely to be influenced by publication bias ( 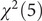 = 11.28, *P* = 0.046).

#### Cognitive function

The effect of exercise on cognitive function in patients with schizophrenia was not significant (*k* = 6; *N* = 47; *g* = 0.01, [95% CI –0.55, 0.58]). None of the moderators assessed were significant (Table 2).

In sensitivity analyses, results showed none of the risk of bias domains moderated effects. The 3PSM likelihood ratio test suggested that these results are likely to be influenced by publication bias ( 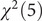 = 14.20, *P* = 0.014).

#### Muscular strength

Our meta-analysis detected a significant effect of exercise on muscular strength among patients with schizophrenia (*k* = 3; *N* = 6; *g* = 0.52, [95% CI 0.22, 0.81]). None of the moderators assessed were significant (Table 2).

In sensitivity analyses, results showed none of the risk of bias domains moderated effects. The 3PSM likelihood ratio test suggested that these results are unlikely to be influenced by publication bias ( 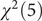 = 3.59, *P* = 0.61).

#### Physical function

There was not a significant effect of exercise on the physical function of patients with schizophrenia (*k* = 8; *N* = 17; *g* = 0.50, [95% CI –0.07, 1.06]). None of the moderators assessed were significant (Table 2).

In sensitivity analyses, results showed none of the risk of bias domains moderated effects. The 3PSM likelihood ratio test suggested that these results are likely to be influenced by publication bias ( 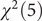 = 13.32, *P* = 0.02).

#### Physical health biomarkers

We did not detect an overall effect of exercise on physical health biomarkers (e.g., lipid profile, fasting glucose, or high blood pressure) among patients with schizophrenia (*k* = 8; *N* = 26; *g* = 0.02, [95% CI –0.24, 0.29]). The type of comparison group significantly moderated these effects ( 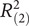 = 74.82%; 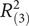 = 0.00%, *P* = 0.036) with exercise tending to improve physical health biomarkers versus occupational therapy (*g* = 0.34, 95% CI [−0.05, 0.73], *k* = 3, *N* = 10), but not in the studies that used treatment as usual (*g* = −0.23, 95% CI [−0.66, 0.18], *k* = 3, *N* = 13) or video games (*g* = −0.15, 95% CI [−0.77, 0.47], *k* = 3, *N* = 3) as a comparison (Table 2).

In sensitivity analyses, blinded outcomes assessors significantly moderated results ( 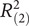 = 0.11, 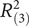 = 0.92, *P* = 0.031) and the studies with blinded evaluators yielded bigger effect sizes (*g* = 0.35, 95% CI [0.06, 0.64], *k* = 3, *N* = 9) than those which evaluators were not blinded to the outcomes assessed (*g* = −0.17, 95% CI [−0.38, 0.04], *k* = 4, *N* = 16). The overall risk of bias score also significantly moderated results ( 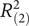 = 0.16, 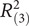 = 1.00, *P* = 0.02) and higher rigor tended toward stronger effects: studies with low overall score of risk of bias demonstrated bigger effect sizes (*g* = 0.48, 95% CI [0.11, 0.84], *k* = 1, *N* = 6) than those which a high risk of bias score were obtained (*g* = −0.13, 95% CI [−0.31, 0.05], *k* = 6, *N* = 19). The 3PSM likelihood ratio test suggested that these results are unlikely to be influenced by publication bias ( 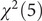 = 2.51, *P* = 0.77).

#### Psychological biomarkers

There was not a significant effect of exercise on the psychological biomarkers (e.g., cortisol, dehydroepiandrosterone, or brain-derived neurotrophic factor) of patients with schizophrenia (*k* = 3; *N* = 4; *g* = −0.12, [95% CI –0.40, 0.17]). None of the moderators assessed were significant (Table 2).

In sensitivity analyses, results showed none of the risk of bias domains moderated effects. The 3PSM likelihood ratio test suggested that these results are unlikely to be influenced by publication bias ( 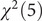 = 4.17, *P* = 0.52).

#### Stress and anxiety

There was not a significant effect of exercise on stress and anxiety of patients with schizophrenia (*k* = 2; *N* = 3; *g* = 0.14, [95% CI –0.11, 0.40]). None of the moderators assessed were significant (Table 2).

In sensitivity analyses, results showed none of the risk of bias domains moderated effects. The 3PSM likelihood ratio test suggested that these results are unlikely to be influenced by publication bias ( 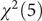 = 9.27, *P* = 0.09).

### Risk of bias and quality of evidence assessments

The results from the PEDro scale indicated that 12 studies had good methodological quality, 13 studies were of fair methodological quality, and 2 studies had very low methodological quality (Table 3). According to the GRADE system, the overall quality of the evidence was low–moderate. The quality for studies investigating the primary outcome of this study, psychopathology, was of moderate quality. The evidence for cardiorespiratory fitness and physical function was of high quality. The quality of evidence for quality of life, anthropometric measurements and composition, ADL and muscular strength outcomes was moderate. For cognitive function and physical health biomarkers the quality of evidence was low and was very low for psychological biomarkers and stress and anxiety indicators (Table 4). Lastly, the PRISMA checklist was checked in the Appendix D of the Supplementary Material.

**Table 3.** Assessment of the methodological quality using the PEDro scale of the articles included in the quantitative analysis.

Criterion	1	2	3	4	5	6	7	8	9	10	11	12	13	14	15	16	17	18	19	20	21	22	23	24	25	26	27	28
Eligibility criteria	Y	N	Y	Y	Y	Y	Y	Y	Y	Y	Y	Y	Y	Y	Y	Y	Y	Y	Y	Y	Y	Y	Y	Y	N	Y	Y	Y
Random allocation	Y	Y	Y	Y	Y	Y	Y	Y	Y	Y	N	Y	Y	Y	Y	Y	Y	Y	Y	Y	Y	Y	Y	Y	Y	N	Y	Y
Concealed allocation	Y	N	Y	N	N	N	N	N	N	N	N	N	N	N	Y	N	Y	Y	Y	Y	Y	Y	Y	Y	Y	N	Y	Y
Baseline comparability	Y	N	Y	Y	Y	Y	Y	Y	Y	Y	Y	Y	Y	Y	Y	Y	Y	Y	Y	Y	Y	Y	Y	Y	Y	Y	Y	Y
Blind subjects	N	N	N	N	N	N	N	N	N	N	N	N	N	N	N	N	N	N	N	N	N	N	N	N	N	N	N	N
Blind therapists	N	N	N	N	N	N	N	N	N	N	N	N	N	N	N	N	N	N	N	N	N	N	N	N	N	N	N	N
Blind assessors	N	N	Y	Y	Y	N	N	N	Y	N	N	N	N	Y	Y	N	N	N	N	Y	Y	Y	N	Y	Y	N	Y	Y
Adequate follow-up	N	N	N	N	N	N	N	N	N	Y	Y	Y	Y	Y	Y	Y	N	Y	N	N	N	Y	N	Y	N	Y	N	N
Intention-to-treat analysis	N	N	Y	N	N	Y	N	N	N	N	N	Y	N	Y	N	N	Y	Y	N	N	Y	N	N	Y	Y	N	N	N
Between group comparisons	Y	Y	Y	Y	Y	Y	Y	Y	Y	Y	Y	Y	Y	Y	Y	Y	Y	Y	Y	Y	Y	Y	Y	Y	Y	Y	Y	Y
Point estimates and variability	Y	N	N	Y	N	Y	N	Y	Y	Y	Y	Y	Y	Y	Y	Y	Y	Y	Y	Y	Y	Y	Y	Y	Y	Y	N	Y
Total	5	2	6	5	4	5	3	4	5	5	4	6	5	7	7	5	6	7	5	6	7	7	5	8	7	4	5	6

*Note:* Y, 1 point; N, 0 point; total, total number of points of each trial. Eligibility criteria item does not contribute to total score.

Included studies: 1, Andersen et al. (2020); 2, Armstrong et al. (2016); 3, Bathia et al. (2017); 4, Battaglia et al. (2013); 5, Beebe et al. (2005); 6, Bredin (2013); 7, Caponnetto et al. (2019); 8, Curcic et al. (2017); 9, E Silva et al. (2015); 10, Heggelund et al. (2011); 11, Heggelund et al. (2012); 12, Ho et al. (2012); 13, Hsu et al. (2016); 14, Ikai et al. (2013); 15, Kaltsatou et al. (2014); 16, Kim et al. (2014); 17, Kimhy et al. (2015); 18, Loh et al. (2016); 19, Marzolini (2009); 20, Pajonk et al. (2010); 21, Rohricht and Priebe (2006); 22, Ryu et al. (2020); 23, Scheewe et al. (2012); 24, Scheewe et al. (2013); 25, Shimada et al. (2019); 26, Shimizu (2017); 27, Svatkova et al. (2015); 28, Varambally et al. (2012).

**Table 4.** Assessment of quality of evidence using GRADE system.

	Domains				
Outcome	1	2	3	4	Quality of evidence
Psychopathology	+	+	−	+	† † †
Quality of life	+	+	−	+	† † †
Anthropometric measurements and composition	+	−	+	+	† † †
Cardiorespiratory fitness	+	+	+	+	† † † †
ADL	−	+	+	+	† † †
Cognitive function	+	+	−	−	† †
Muscular strength	+	−	+	+	† † †
Physical function	+	+	+	+	† † † †
Physical health biomarkers	+	+	−	−	† †
Psychological biomarkers	−	−	−	+	†
Stress and anxiety	−	−	−	+	†

1, risk of bias; 2, imprecision; 3, inconsistency; 4, indirectness; † † † †, high quality; † † †, moderate quality; † †, low quality; †, very low quality.

## Discussion

This review of 28 studies (1,460 participants) was set to examine the effects of exercise on psychopathology (primary outcome) and other commonly reported clinical outcomes in patients diagnosed with schizophrenia. Pooled effect estimates across all psychopathology outcomes (14 studies; 828 participants) showed that supervised exercise interventions significantly improve psychopathology in patients with schizophrenia. Effect sizes moderately varied between studies. Our moderation analyses showed that exercise was consistently superior to the provision of usual treatment and occupational therapy. The positive effects of exercise on psychopathology were stronger in outpatients than those who were institutionalized. Remarkably, the observed benefits were independent of the type of exercise performed.

Our results are broadly consistent with existing literature [14, 16–18, 36] and demonstrate the positive effects of exercise for psychopathology outcomes in patients with schizophrenia. Our findings may be partially explained by the capacity of exercise to reduce oxidative stress [37] and stimulate the release of brain-derived neurotrophic factor [38] in this population. Nonetheless, the effect size from our meta-analysis (*g* = 0.28) was smaller than that found by Firth et al. [17] and Dauwan et al. [14] (*g* = 0.72 and 0.39, respectively). Possibly, these differences are due to the advantageous use of multilevel meta-analytic techniques in our study, which also produced more precise (i.e., narrower) confidence intervals. It is also worth noting that the effect size found in our meta-analysis was comparable to the effect of common antipsychotic drugs [39, 40]. As a novelty, our moderation analysis indicated that the psychopathology benefits of exercise were greater in outpatients. Several factors may account for this observation. First, the lack of freedom [41] and increase in medication dosage [42] often observed in mental health institutions may increase the stigma of patients with schizophrenia [42]. This stigmatization may result in exacerbated negative symptoms [42] and may prevent patients from engaging in healthy lifestyles, including exercise [43]. Conversely, outpatients have better functionality and enjoy the freedom inpatients do not, which may also result in healthier lifestyle choices [44, 45], possibly supported by family and friends [42]. In addition, adherence to exercise programs is lower among inpatients compared with outpatients [46], which ultimately may undermine the utility of exercise-based strategies to treat psychopathology in patients with schizophrenia that are institutionalized. Therefore, finding strategies to increase the adherence to exercise programs is crucial for patients with schizophrenia [47], particularly among inpatients [47].

Our results also suggest that exercise may be efficacious to improve muscular strength and ADL in patients with schizophrenia, which is consistent with previous meta-analyses and single trials [14, 15, 17]. Although not surprising, this finding is relevant, as muscle loss is often observed in patients with schizophrenia, particularly among those with impacted psychopathology [48]. These results were robust to the covariates explored in this meta-analysis. Consistently, our estimates also suggest that exercise can improve physical functioning and quality of life of this population group [14]. Intriguingly, the benefits of exercise for quality of life were larger in inpatients than in outpatients. A partial explanation for this finding could be that the addition of an exercise routine may be perceived by patients as something novel in a very controlled environment, which could enhance their quality of life [49]. Drop-out rates have been documented to be lower in acute settings [50], which could also influence the results. Lastly, patients in acute care tend to depict lower levels of self-perceived health and may therefore have more room for improvement [51]. We found that the type of exercise performed did also moderate the results on quality of life. Consistent with previous studies, aerobic exercise and HIIT were more effective to improve quality of life, but yoga and low-intensity aerobic exercise were not, showing the latter a negative association with this variable. It may be possible that a certain intensity is required to elicit significant self-perceived health benefits [52]. Nonetheless, these negative results associated with yoga and low-intensity aerobic exercise may just be an artifact that reflects the low number of studies exploring these two exercise modalities in our review. Similarly, cardiorespiratory fitness improved more in outpatients, possibly because the majority of studies in this setting used HIIT or aerobic exercise and the number of studies in inpatients were low. Other outcomes in this meta-analysis were not significant, including anthropometric parameters, body composition, cognition, biomarkers, and stress. Factors such as unhealthy lifestyle habits commonly observed in patients with schizophrenia [10] or medication (e.g., antipsychotics) [50] coupled with some schizophrenia manifestations such as apathy, lack of motivation, or cognitive deficits [13] may partially account for these observations. Also, several studies have shown the lower levels of physical activity in people with schizophrenia compared with the general population [51], which may contribute to the observed lack of significant results in our study. Our results in cognition and stress contrast with previous findings [15, 17, 18], although we found the heterogeneity at study level (i.e., level 3) was high. However, none of the moderators tested was significant. Future meta-analysis may want to explore other moderators in a multilevel analytical framework to tear apart the effects of exercise on depression and cognition among patients with schizophrenia.

Our study has several important strengths. First, we used multilevel meta-analytic techniques, which allowed us to effectively account for the nested nature of effect sizes originated from the same studies, thereby reducing estimation bias [23]. Another key strength was that we explored important moderators relevant to clinical practice (i.e., clinical setting, type of exercise, comparison group, and the existence of post-intervention follow-up) which may help to inform the decision-making process of using exercise as co-adjuvant therapy in patients with schizophrenia. Lastly, we assessed several clinically relevant outcomes in the same context, which provides a comprehensive picture of the utility of exercise in this population group.

There are nevertheless some study limitations that need to be considered when interpreting the results. First, six of the studies included in our review were of low or very low quality. Nonetheless, the sensitivity analysis performed indicated results were robust to study quality assessment. Second, the heterogeneity in outcomes measures in the included studies prevented us from accurately determining clinically meaningful changes in this meta-analysis, which may limit the applicability of our results. Third, physical function and physical health biomarkers outcomes showed considerable heterogeneity at between-study level which was not fully explained by our moderators. Women were underrepresented in the included studies, which may limit the generalization of our results. Nonetheless, the prevalence of schizophrenia is lower among women [53, 54]. Moreover, the majority of studies described interventions based on aerobic exercise, which may have downplayed other promising exercise types such as resistance exercise or concurrent (i.e., combined aerobic and strength exercises). Lastly, several outcomes (i.e., body anthropometric and composition, ADL, cognitive function, physical function and stress and anxiety outcomes) were likely to be influenced by publication bias. Adherence to exercise is problematic in patients with schizophrenia [52]. Future studies need to consider additional strategies to improve this aspect in order to fully realize the potential of exercise interventions in patients with schizophrenia [55, 56].

In conclusion, our meta-analysis has provided robust evidence that supervised exercise is effective to improve the psychopathology of patients with schizophrenia. We demonstrated that exercise could also be useful to treat other relevant outcomes in this population group (i.e., quality of life, ADL, muscular strength, and physical function), which may help alleviate some of the most pressing issues associated with schizophrenia, such as the side effects of antipsychotics or the lack of adherence to medication. Outpatients seemed to have a greater benefit both in terms of quality of life and cardiorespiratory fitness, while inpatients showed greater improvements in their quality of life, with HIIT and aerobic exercise modalities presenting the greatest effects in this outcome. Together, our findings provide useful insights to inform the design of effective exercise interventions for patients with schizophrenia, which importantly contribute to build a solid evidence base for psychoeducation material that may facilitate the uptake of exercise in this population and related psychotic disorders. In light of the current evidence, clinicians and decision-makers should consider exercise as part of the clinical care pathway of patients with schizophrenia.

## Data Availability

All data required to reproduce the analyses included in this meta-analysis are provided through public repository access (https://github.com/dgalgom).

## Supplementary material

For supplementary material accompanying this paper visit http://doi.org/10.1192/j.eurpsy.2023.24. click here to view supplementary material
