# Supplementary material for: Exercise to treat psychopathology and other clinical outcomes in schizophrenia: A systematic review and meta-analysis
Source: Eur Psychiatry. 2023 Apr 25;66(1):e40. doi: 10.1192/j.eurpsy.2023.24 (PMC10305321; doi:10.1192/j.eurpsy.2023.24)
Supplement: Supplementary file 1 [file S092493382300024Xsup001.docx]

**Appendix D “Included studies”**

1. [Andersen E, Bang-Kittilsen G, Bigseth TT, et al. Effect of high-intensity interval training on cardiorespiratory fitness, physical activity and body composition in people with schizophrenia: a randomized controlled trial. *BMC Psychiatry*. 2020;20(1):425. doi:](http://paperpile.com/b/kOfLWv/qKnTo)[10.1186/s12888-020-02827-2](http://dx.doi.org/10.1186/s12888-020-02827-2)

2. [Armstrong HF, Bartels MN, Paslavski O, et al. The impact of aerobic exercise training on cardiopulmonary functioning in individuals with schizophrenia. *Schizophrenia Research*. 2016;173(1-2):116-117. doi:](http://paperpile.com/b/kOfLWv/jHVF)[10.1016/j.schres.2016.03.009](http://dx.doi.org/10.1016/j.schres.2016.03.009)

3. [Bhatia T, Mazumdar S, Wood J, et al. A randomised controlled trial of adjunctive yoga and adjunctive physical exercise training for cognitive dysfunction in schizophrenia. *Acta Neuropsychiatr*. 2017;29(2):102-114. doi:](http://paperpile.com/b/kOfLWv/NfYz)[10.1017/neu.2016.42](http://dx.doi.org/10.1017/neu.2016.42)

4. [Battaglia G, Alesi M, Inguglia M, et al. Soccer practice as an add-on treatment in the management of individuals with a diagnosis of schizophrenia. *Neuropsychiatr Dis Treat*. 2013;9:595-603. doi:](http://paperpile.com/b/kOfLWv/E7XD)[10.2147/NDT.S44066](http://dx.doi.org/10.2147/NDT.S44066)

5. [Beebe LH, Tian L, Morris N, Goodwin A, Allen SS, Kuldau J. Effects of exercise on mental and physical health parameters of persons with schizophrenia. *Issues Ment Health Nurs*. 2005;26(6):661-676. doi:](http://paperpile.com/b/kOfLWv/s9Xi)[10.1080/01612840590959551](http://dx.doi.org/10.1080/01612840590959551)

6. [Bredin SSD, Warburton DER, Lang DJ. The health benefits and challenges of exercise training in persons living with schizophrenia: a pilot study. *Brain Sci*. 2013;3(2):821-848. doi:](http://paperpile.com/b/kOfLWv/7kbF)[10.3390/brainsci3020821](http://dx.doi.org/10.3390/brainsci3020821)

7. [Caponnetto P, Auditore R, Maglia M, Pipitone S, Inguscio L. Psychological wellness, yoga and quality of life in patients affected by schizophrenia spectrum disorders: A pilot study. *Ment Illn*. 2019;11(1):8003. doi:](http://paperpile.com/b/kOfLWv/v3vj)[10.4081/mi.2019.8003](http://dx.doi.org/10.4081/mi.2019.8003)

8. [Curcic D, Stojmenovic T, Djukic-Dejanovic S, et al. Positive impact of prescribed physical activity on symptoms of schizophrenia: randomized clinical trial. *Psychiatr Danub*. 2017;29(4):459-465. doi:](http://paperpile.com/b/kOfLWv/Ru0Y)[10.24869/psyd.2017.459](http://dx.doi.org/10.24869/psyd.2017.459)

9. [Andrade e Silva B, Cassilhas RC, Attux C, et al. A 20-week program of resistance or concurrent exercise improves symptoms of schizophrenia: results of a blind, randomized controlled trial. *Braz J Psychiatry*. 2015;37(4):271-279. doi:](http://paperpile.com/b/kOfLWv/G5yo)[10.1590/1516-4446-2014-1595](http://dx.doi.org/10.1590/1516-4446-2014-1595)

10. [Heggelund J, Nilsberg GE, Hoff J, Morken G, Helgerud J. Effects of high aerobic intensity training in patients with schizophrenia: a controlled trial. *Nord J Psychiatry*. 2011;65(4):269-275. doi:](http://paperpile.com/b/kOfLWv/gmFo)[10.3109/08039488.2011.560278](http://dx.doi.org/10.3109/08039488.2011.560278)

11. [Heggelund J, Morken G, Helgerud J, Nilsberg GE, Hoff J. Therapeutic effects of maximal strength training on walking efficiency in patients with schizophrenia - a pilot study. *BMC Res Notes*. 2012;5(1):344. doi:](http://paperpile.com/b/kOfLWv/jpgB)[10.1186/1756-0500-5-344](http://dx.doi.org/10.1186/1756-0500-5-344)

12. [Ho SS, Dhaliwal SS, Hills AP, Pal S. The effect of 12 weeks of aerobic, resistance or combination exercise training on cardiovascular risk factors in the overweight and obese in a randomized trial. *BMC Public Health*. 2012;12:704. doi:](http://paperpile.com/b/kOfLWv/BBP8)[10.1186/1471-2458-12-704](http://dx.doi.org/10.1186/1471-2458-12-704)

13. [Hsu C-C, Liang C-S, Tai Y-M, Cheng S-L. Incongruent changes in heart rate variability and body weight after discontinuing aerobic exercise in patients with schizophrenia. *Int J Psychophysiol*. 2016;109:132-137. doi:](http://paperpile.com/b/kOfLWv/oeVM)[10.1016/j.ijpsycho.2016.08.011](http://dx.doi.org/10.1016/j.ijpsycho.2016.08.011)

14. [Ikai S, Uchida H, Suzuki T, Tsunoda K, Mimura M, Fujii Y. Effects of yoga therapy on postural stability in patients with schizophrenia-spectrum disorders: a single-blind randomized controlled trial. *J Psychiatr Res*. 2013;47(11):1744-1750. doi:](http://paperpile.com/b/kOfLWv/lUnH)[10.1016/j.jpsychires.2013.07.017](http://dx.doi.org/10.1016/j.jpsychires.2013.07.017)

15. [Kaltsatou ACH, Kouidi EI, Anifanti MA, Douka SI, Deligiannis AP. Functional and psychosocial effects of either a traditional dancing or a formal exercising training program in patients with chronic heart failure: a comparative randomized controlled study. *Clin Rehabil*. 2014;28(2):128-138.](http://paperpile.com/b/kOfLWv/MRh8) <https://journals.sagepub.com/doi/abs/10.1177/0269215513492988?casa_token=F24xGAANjgYAAAAA:VDc4Ggl2HbIBkPxXXeC3drGIZZ0GAwhQlvHLdkmX_gbkn_fKtO5JwavfvYpNDUdsp2Zo-11KNynjoA>

16. [Kim H-J, Song B-K, So B, Lee O, Song W, Kim Y. Increase of circulating BDNF levels and its relation to improvement of physical fitness following 12 weeks of combined exercise in chronic patients with schizophrenia: a pilot study. *Psychiatry Res*. 2014;220(3):792-796. doi:](http://paperpile.com/b/kOfLWv/m9QL)[10.1016/j.psychres.2014.09.020](http://dx.doi.org/10.1016/j.psychres.2014.09.020)

17. [Kimhy D, Vakhrusheva J, Bartels MN, et al. The Impact of Aerobic Exercise on Brain-Derived Neurotrophic Factor and Neurocognition in Individuals With Schizophrenia: A Single-Blind, Randomized Clinical Trial. *Schizophr Bull*. 2015;41(4):859-868. doi:](http://paperpile.com/b/kOfLWv/eDaA)[10.1093/schbul/sbv022](http://dx.doi.org/10.1093/schbul/sbv022)

18. [Loh SY, Abdullah A, Abu Bakar AK, Thambu M, Nik Jaafar NR. Structured Walking and Chronic Institutionalized Schizophrenia Inmates: A pilot RCT Study on Quality of Life. *Glob J Health Sci*. 2015;8(1):238-248. doi:](http://paperpile.com/b/kOfLWv/1vxk)[10.5539/gjhs.v8n1p238](http://dx.doi.org/10.5539/gjhs.v8n1p238)

19. [Marzolini S, Jensen B, Melville P. Feasibility and effects of a group-based resistance and aerobic exercise program for individuals with severe schizophrenia: A multidisciplinary approach. *Mental Health and Physical Activity*. 2009;2(1):29-36. doi:](http://paperpile.com/b/kOfLWv/EnhB)[10.1016/j.mhpa.2008.11.001](http://dx.doi.org/10.1016/j.mhpa.2008.11.001)

20. [Pajonk F-G, Wobrock T, Gruber O, et al. Hippocampal plasticity in response to exercise in schizophrenia. *Arch Gen Psychiatry*. 2010;67(2):133-143. doi:](http://paperpile.com/b/kOfLWv/gLL7)[10.1001/archgenpsychiatry.2009.193](http://dx.doi.org/10.1001/archgenpsychiatry.2009.193)

21. [Röhricht F, Priebe S. Effect of body-oriented psychological therapy on negative symptoms in schizophrenia: a randomized controlled trial. *Psychol Med*. 2006;36(5):669-678. doi:](http://paperpile.com/b/kOfLWv/GVRz)[10.1017/S0033291706007161](http://dx.doi.org/10.1017/S0033291706007161)

22. [Ryu J, Jung JH, Kim J, et al. Outdoor cycling improves clinical symptoms, cognition and objectively measured physical activity in patients with schizophrenia: A randomized controlled trial. *J Psychiatr Res*. 2020;120:144-153. doi:](http://paperpile.com/b/kOfLWv/khTd)[10.1016/j.jpsychires.2019.10.015](http://dx.doi.org/10.1016/j.jpsychires.2019.10.015)

23. [Scheewe TW. Effects of exercise therapy on cardiovascular fitness and the metabolic syndrome in schizophrenia: a randomized clinical trial. *Schizophrenia Research*. 2012;136:S47. doi:](http://paperpile.com/b/kOfLWv/ZvqS)[10.1016/s0920-9964(12)70172-x](http://dx.doi.org/10.1016/s0920-9964(12)70172-x)

24. [Scheewe TW, van Haren NEM, Sarkisyan G, et al. Exercise therapy, cardiorespiratory fitness and their effect on brain volumes: a randomised controlled trial in patients with schizophrenia and healthy controls. *Eur Neuropsychopharmacol*. 2013;23(7):675-685. doi:](http://paperpile.com/b/kOfLWv/4Jzm)[10.1016/j.euroneuro.2012.08.008](http://dx.doi.org/10.1016/j.euroneuro.2012.08.008)

25. [Shimada T, Ito S, Makabe A, Yamanushi A, Takenaka A, Kobayashi M. Aerobic exercise and cognitive functioning in schizophrenia: A pilot randomized controlled trial. *Psychiatry Res*. 2019;282:112638. doi:](http://paperpile.com/b/kOfLWv/rO6C)[10.1016/j.psychres.2019.112638](http://dx.doi.org/10.1016/j.psychres.2019.112638)

26. [Shimizu N, Umemura T, Matsunaga M, Hirai T. An interactive sports video game as an intervention for rehabilitation of community-living patients with schizophrenia: A controlled, single-blind, crossover study. *PLoS One*. 2017;12(11):e0187480. doi:](http://paperpile.com/b/kOfLWv/TPrE)[10.1371/journal.pone.0187480](http://dx.doi.org/10.1371/journal.pone.0187480)

27. [Svatkova A, Mandl RCW, Scheewe TW, Cahn W, Kahn RS, Hulshoff Pol HE. Physical Exercise Keeps the Brain Connected: Biking Increases White Matter Integrity in Patients With Schizophrenia and Healthy Controls. *Schizophr Bull*. 2015;41(4):869-878. doi:](http://paperpile.com/b/kOfLWv/xxwK)[10.1093/schbul/sbv033](http://dx.doi.org/10.1093/schbul/sbv033)

28. [Varambally S, Gangadhar BN, Thirthalli J, et al. Therapeutic efficacy of add-on yogasana intervention in stabilized outpatient schizophrenia: Randomized controlled comparison with exercise and waitlist. *Indian J Psychiatry*. 2012;54(3):227-232. doi:](http://paperpile.com/b/kOfLWv/jhsP)[10.4103/0019-5545.102414](http://dx.doi.org/10.4103/0019-5545.102414)
